# N6-methylandenosine-related lncRNAs have the prognostic predictive ability for patients with endometrial cancer

**DOI:** 10.1097/MD.0000000000046657

**Published:** 2025-12-19

**Authors:** Jiajie She, Ling Shuai, Danna Su, Xiaofeng Ye, Qingyu Hu, Dongdong Li, Yan Guo, Xuemin Liu, Ruiying Dia

**Affiliations:** a Department of Ocean Science and Hong Kong Branch of the Southern Marine Science and Engineering Guangdong Laboratory (Guangzhou), The Hong Kong University of Science and Technology, Clear Water Bay, Hong Kong, China; b Southern Marine Science and Engineering Guangdong Laboratory (Guangzhou), Guangzhou, China; c The First Affiliated Hospital of Shenzhen University, Reproductive Medicine Centre, Shenzhen Second People’s Hospital, Shenzhen, China; d South China Hospital of Shenzhen University, Department of Obstetrics and Gynecology, Shenzhen, China.

**Keywords:** ceRNA network, endometrial carcinoma (EC), immune cell infiltration, long noncoding RNA (lncRNA), N6-methylandenosine (m6A), prognostic signature

## Abstract

Endometrial carcinoma (EC) ranks as the fourth most common cancer among women, with increasing morbidity and mortality rates in recent years. Identifying reliable biomarkers for prognosis and therapeutic targets is crucial for improving the outcomes of EC patients. In this study, we identified 1560 m6A-related lncRNAs using Pearson correlation coefficient based on the cancer genome atlas datasets and GENCODE annotation. Through univariate Cox regression analysis, 187 m6A-related lncRNAs were found to be related to EC prognosis. Using least absolute shrinkage and selection operator Cox analysis, we constructed an m6A-related lncRNA prognostic signature (m6A-LPS) comprising 12 m6A-related lncRNAs. The m6A-LPS demonstrated robust prognostic ability, and the nomogram incorporating m6A-LPS, age, and pathological grade effectively predicted overall survival of EC patients. Furthermore, EC patients in the low-risk group exhibited higher immune cell infiltration and lower tumor purity. A competing endogenous RNA (ceRNA) network was constructed to elucidate the potential function of these m6A-related prognostic lncRNAs. Our study establishes the m6A-LPS as a stable and reliable prognostic tool for EC patients. These findings may contribute to the identification of novel biomarkers and therapeutic targets, offering insights into the molecular mechanisms underlying EC pathogenesis.

## 1. Introduction

Endometrial carcinoma (EC) is one of the most prevalent malignancies among women, with rising morbidity and mortality worldwide in recent decades.^[1]^ According to GLOBOCAN, there were nearly 382,000 new cases and 89,900 deaths in 2018.^[2]^ While over 70% of EC patients are diagnosed at an early stage, those with advanced-stage disease face a poor prognosis, with a 5-year survival rate of approximately 16%.^[3]^ Therefore, it is crucial and urgent for the prognosis of EC to identify potential reliable biomarkers as therapeutic targets.

As the most abundant epigenetic modification of mRNA and long noncoding RNA (lncRNA), N6-methylandenosine (m6A) modification plays crucial role in RNA stability, splicing, and translation.^[4–7]^ Recent studies have implicated m6A modifications in the pathogenesis of EC. For instance, METTL14 (an m6A methyltransferase) regulates AKT activity, promoting the proliferation and tumorigenicity of EC.^[8]^ FTO (an m6A demethylase) promotes EC metastasis through activating the WNT signaling pathway.^[9]^ Another m6A demethylase ALKBH5 regulates IGF1R expression, contributing to the proliferation and tumorigenicity of EC.^[10]^ The m6A reader YTHDF2 mediates the degradation of lncRNA FENDRR, thus promoting cell proliferation in EC.^[11]^ A previous study indicated that YTHDF2 negatively regulated the IRS1’s expression, thus inhibiting the tumorigenicity of EC.^[12]^ Another m6A reader, IGF2BP1, regulates the stability of PEG10 mRNA and thus promoted the cell proliferation and progression of EC.^[13]^ Furthermore, some m6A regulators have been shown to be associated with the overall survival (OS) rate of EC patients.^[14]^

In addition to m6A modification, long noncoding RNAs (lncRNAs) are crucial in EC pathogenesis.^[15]^ For example, HAND2-AS1 downregulated the expression of neuromedin U and thus inhibit the invasion and metastasis of EC.^[16]^ DLEU2 activates HK2-driven epithelial-mesenchymal transition, suppressing EC proliferation.^[17]^ MIR210HG promotes EC progression by inhibiting Wnt and TGF-β signaling pathways.^[18]^ High BLACAT1 expression correlates with poor OS in EC patients.^[19]^ However, few studies have explored the regulatory mechanisms of m6A modifications by lncRNAs in EC, and the relationship between m6A-related lncRNAs and EC progression remains unclear. Therefore, it’s crucial to understand how m6A-related lncRNAs contribute to EC pathogenesis for identifying potential biomarkers as therapeutic targets.

In this study, we performed bioinformatics and statistical analyses to determine the prognostic role of m6A-related lncRNAs in EC patients using the TCGA dataset. We established an m6A-related lncRNA prognostic signature (m6A-LPS) for the prediction of OS in EC patients and explored its association with immune response. These findings indicated that these m6A-related prognostic lncRNAs had prognostic predictive ability for patients with Endometrial Cancer, which could act as potential biomarkers for the prognosis of EC.

## 2. Methods

### 2.1. Collection and processing of EC-related dataset and m6A-related lncRNAs

Through the Cancer Genome Atlas (TCGA) database and UCSC Xena, we obtained a TCGA dataset that included 583 patients. Expression (normalized FPKM value) data was extracted for subsequent analyses. To reduce statistical bias, we excluded those EC patients with missing OS values or OS < 1 month. Finally, 35 normal and 525 tumor samples were included in the subsequent analysis. The clinical characteristics of these samples were shown in Table S1, Supplemental Digital Content, https://links.lww.com/MD/Q957. Furthermore, the expression matrix of 21 m6A-related genes (“writers”: METTL3, METTL14, METTL16, WTAP, VIRMA, RBM15, RBM15B, and ZC3H13; “erasers”: FTO and ALKBH5; “readers”: YTHDC1, YTHDC2, IGF2BP1, IGF2BP2, IGF2BP3, YTHDF1, YTHDF2, YTHDF3, HNRNPC, HNRNPA2B1, and RBMX) was extracted from the TCGA dataset. The annotation information (GRCH38) of long noncoding RNAs (lncRNAs) was downloaded from the GENCODE database. After filtering gene types, we identified 14,032 lncRNAs. Using Pearson correlation coefficient analysis, we found 1560 m6A-related lncRNAs and 13,181 m6A-lncRNA pairs with a threshold of |Pearson correlation coefficient| > 0.5 and *P* < .001).

### 2.2. Survival analysis

Based on clinical information, univariate Cox regression analysis was performed to screen for m6A-related prognostic lncRNAs. Based on the R package “glmnet,”^[20]^ we established an m6A-related lncRNA prognostic signature (m6A-LPS) for EC patients with the least absolute shrinkage and selection operator (LASSO) Cox regression (10-fold cross-validation).^[21]^ The formula for calculating risk scores is as follows:


$$\begin{array}{l} Risk\;score=\overset{n}{\underset{i=1}{\sum }}Coef_{i}*x_{i} \end{array}$$

where $Coef_{i}$ is the coefficient, $x_{i}$ is the expression level of each m6A-related lncRNA. EC patients were divided into high-risk and low-risk groups based on the median risk score. Based on the R package (“survival,” “survminer” and “ggrisk”), survival analysis was conducted between these 2 groups.

### 2.3. Independent prognostic analysis of m6A-related lncRNAs and validation

To investigate whether clinical characteristics had an impact on the prognostic role of m6A-related lncRNAs, univariate independent prognostic analysis was performed in terms of age and pathological grade using the Kaplan–Meier “survival” package. Univariate and multivariate Cox analyses were performed to explore the prognostic predictive role of m6A-related lncRNAs in EC patients. The receiver operating characteristic (ROC) evaluation curve plotted with “survROC” package was used to validate the prediction ability of m6A-LPS. Furthermore, the nomogram (based on m6A-LPS) established by the “rms,” “foreign” and “survival” packages was used to evaluate the prognostic predictive role of the clinical characteristics and m6A-related lncRNAs.

### 2.4. Tumor immune microenvironment analysis between different risk groups

The CIBERSORT algorithm calculates the putative proportion of immune and stromal cell fractions from gene expression matrices.^[22]^ To estimate the relative abundance of tumor-infiltrating immune cells (TIICs) in tumor samples, 22 sorted immune cells (LM22) were used as a reference set. According to the algorithm using the “estimate” packages, we calculated the score of the immune and stromal cells in the tumor microenvironment (TME). The higher the score, the greater the ratio of the corresponding proportion in the TME. Combined with the risk status, differences in TIICs between the high-risk and low-risk groups were identified.

### 2.5. Bioinformatic analysis

The “rgl” package was used to perform principal component analysis (PCA) for high-risk and low-risk groups, which was plotted with the “factoextra” package. According to the m6A-LPS, the risk scores of all samples were calculated. Based on the “limma” package,^[23]^ the differentially expressed genes (DEGs) between high-risk and low-risk groups in the TCGA dataset were identified with a threshold of |log2(Fold Change)| > 1 and *P* < .05. To construct the lncRNA-miRNA-mRNA (ceRNA) network, the target miRNAs of 12 m6A-related prognostic lncRNAs were extracted from the miRcode database^[24]^ and the miRNA targets were commonly identified from^[25]^ and TargetScan databases.^[26]^ The Metascape database^[27]^ was used to perform pathway and process enrichment analysis for DEGs and target mRNAs. The ceRNA network was constructed and visualized using Cytoscape software.^[28]^ Furthermore, the heatmap plot and box plot were plotted with the “pheatmap” and “ggplot2” packages.

### 2.6. Statistical analysis

The risk scores calculated using the m6A-LPS were compared between different groups or subgroups based on clinical characteristics including age (<60 or≥60 years) and pathological grade (G1, G2, G3 and high-grade) using the t-test. The Chi-square test was used to compare the clinical characteristics between the high and low-risk groups. Based on the expression profiles of m6A-related lncRNAs, the OS of EC patients was compared between different risk groups or clinical subgroups using Kaplan–Meier curves. Furthermore, univariate and multivariate Cox regression analysis were used to evaluate the independent prognostic value of m6A-LPS in the OS of EC patients. The nomogram was used to evaluate the prognostic predictive accuracy of m6A-LPS.

## 3. Results

### 3.1. Identification of m6A-related prognostic lncRNAs in EC Patients

**Figure 1.**
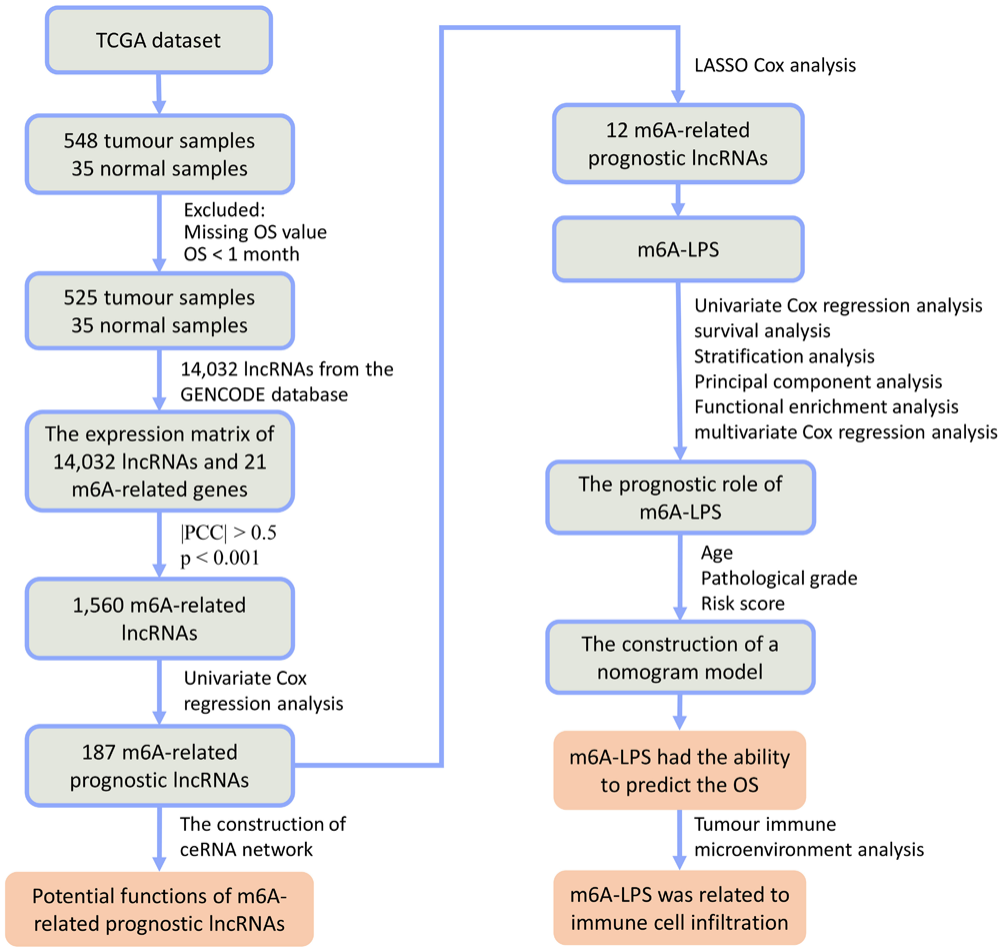
The flow chart of the whole study.

Based on the GENCODE database, we identified 14,032 lncRNAs for subsequent analysis. The expression matrixes of these lncRNAs and 21 m6A-related genes were extracted from the TCGA datasets for the Pearson correlation analysis, respectively. The m6A-related lncRNAs were identified by the correlation between the expression level of these lncRNAs and 21 m6A-related genes (| correlation coefficient | > 0.5 and *P* < .001). Based on the Pearson correlation analysis, we identified 1560 lncRNAs that were significantly correlated with m6A-related genes (Table S2, Supplemental Digital Content, https://links.lww.com/MD/Q957). To screen m6A-related prognostic lncRNAs, univariate Cox regression analysis was conducted through the integration of the prognostic information. We found that 187 m6A-related lncRNAs were significantly correlated with the OS of EC patients (*P* < .005). The expression heatmap of these m6A-related lncRNAs was presented in Figure S1, Supplemental Digital Content, https://links.lww.com/MD/Q956. The results of univariate Cox regression analysis of these 187 m6A-related lncRNAs were shown in Table S3, Supplemental Digital Content, https://links.lww.com/MD/Q957. A flow chart of this study was shown in Figure 1.

### 3.2. Construction of the m6A-LPS based on the LASSO Cox analysis

**Figure 2.**
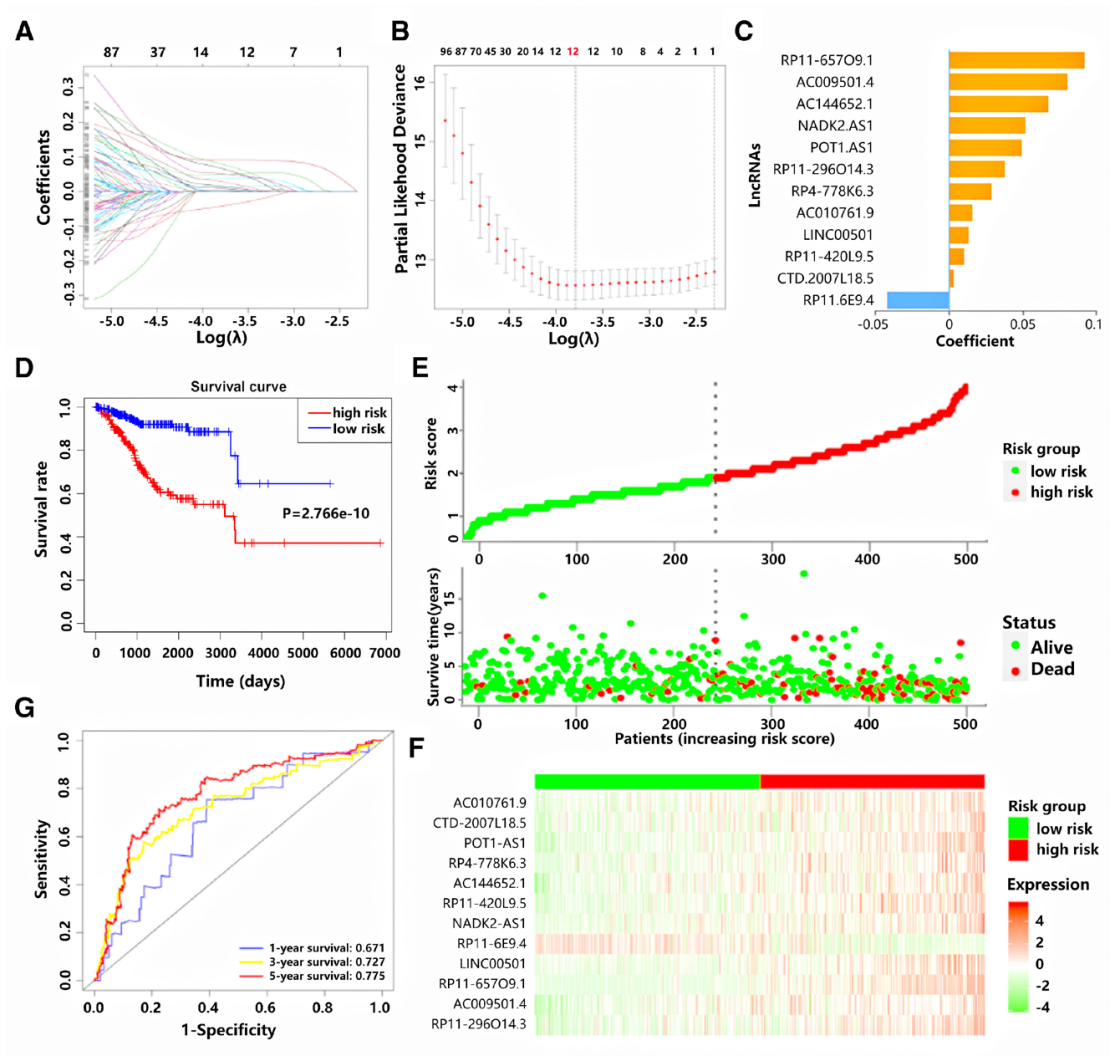
Construction of the m6A-LPS. (A–C) The minimum criteria (A and B) and coefficients (C) were calculated through the LASSO Cox regression analysis. (D) Kaplan–Meier survival curves revealed that patients in low-risk group had better OS than those in the high-risk group. (E) The distributions of risk scores and survival status of EC patients. (F) The heatmap of twelve m6A-related prognostic lncRNAs in different risk groups. (G) The ROC evaluation curves for the prediction of the 1, 3 and 5-yr survival rate. LASSO = least absolute shrinkage and selection operator, lncRNA = long noncoding RNA, m6A-LPS = m6A-related lncRNA prognostic signature, ROC = receiver operating characteristic.

Based on the 187 m6A-related prognostic lncRNAs, the LASSO regression analysis was performed to establish the m6A-LPS for predicting the OS of EC patients. The minimum criteria and coefficients were calculated (Fig. 2A and B) and m6A-LPS was constructed, which included 12 m6A-related lncRNAs. The risk scores of all patients were calculated based on the coefficients of 12 lncRNAs (Fig. 2C). All patients were divided into high and low-risk groups based on the median value of the risk score. The Kaplan–Meier survival curves showed that the EC patients in the low-risk group had higher survival rate, indicating better clinical outcomes (Fig. 2D). The distributions of risk score and survival status were visualized in Figure 2E. The heatmap of 12 m6A-related prognostic lncRNAs revealed that the expression level of RP11-6E9.4 was higher in low-risk group than high-risk group, whereas the opposite trend was observed for other m6A-related prognostic lncRNAs (Fig. 2F). The ROC curve indicated that the constructed m6A-LPS had the ability to forecast the OS of EC patients in the TCGA datasets (Fig. 2G) (AUC value of 1-year OS, 3-year OS and 5-year OS were 0.671, 0.727 and 0.775, respectively).

### 3.3. Univariate Cox regression analysis and survival analysis for 12 m6A-related prognostic lncRNAs

**Figure 3.**
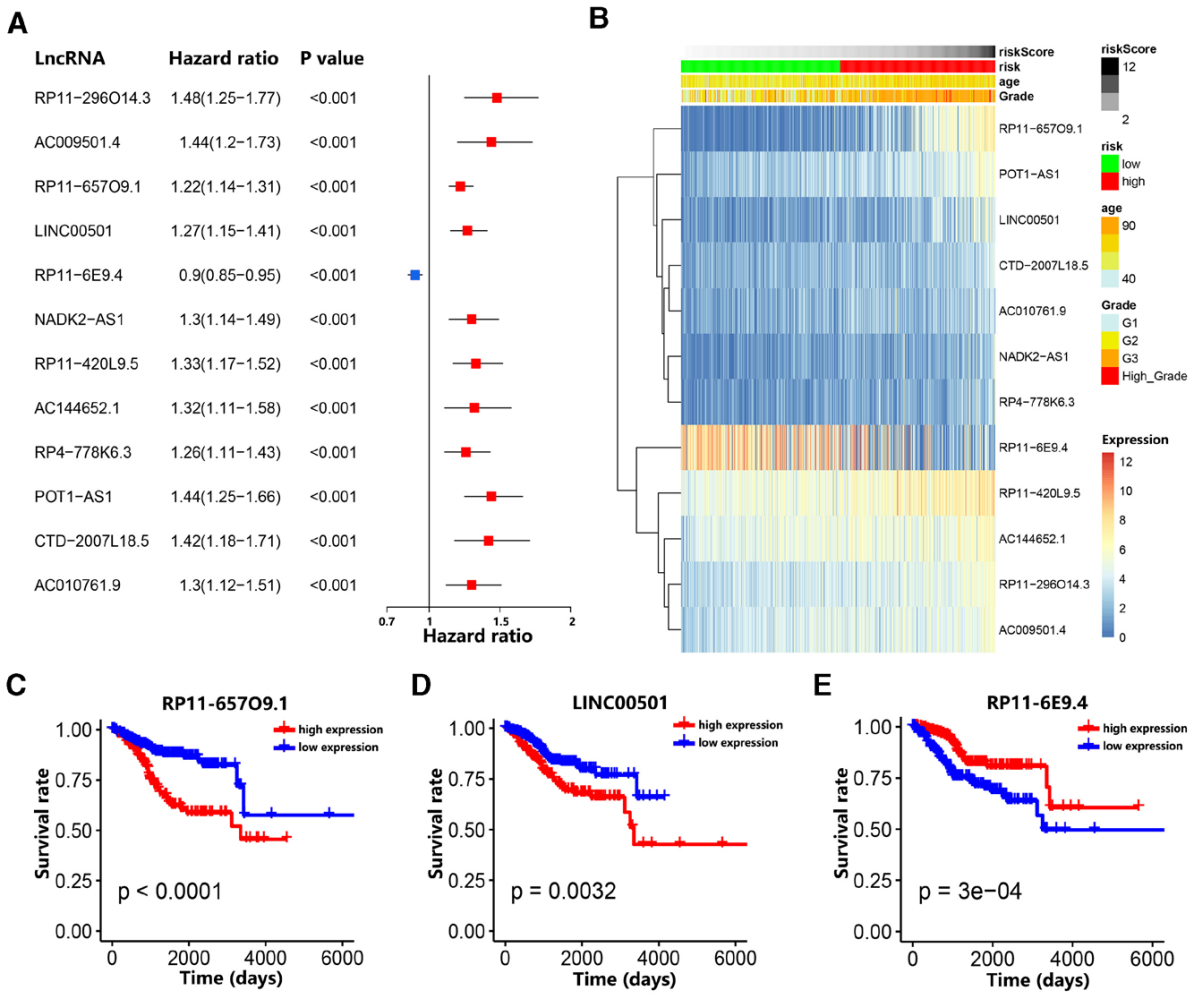
The relationship between twelve m6A-related prognostic lncRNAs and risk score, OS. (A) The forest plot of the predictive ability of twelve m6A-related prognostic lncRNAs. (B) The heatmap of the relationship between the expression levels of twelve m6A-related prognostic lncRNAs and clinical characteristics. (C–E) Kaplan–Meier curves showed that EC patients with low expression levels of 3 m6A-related prognostic lncRNAs had better OS. EC = endometrial carcinoma, lncRNA = long noncoding RNA, OS = overall survival.

To evaluate the prognostic predictive value of 12 m6A-related lncRNAs included in the m6A-LPS, we performed univariate Cox regression analysis. The visualization of the forest plot suggested that RP11-6E9.4 was a protective factor (Hazard ratio < 1), while other m6A-related lncRNAs were risk factors (Hazard ratio > 1) in EC patients (Fig. 3A). The heatmap showed that the expression level of RP11-6E9.4 decreased with increasing risk score, whereas the expression level of other m6A-related lncRNAs increased with increasing risk scores. Furthermore, the expression level of these m6A-related lncRNAs was also associated with some clinical characteristics of EC patients, including age and pathological grade (Fig. 3B). Through the Kaplan–Meier survival curves, we found that high expression of RP11-6E9.4 and low expression of RP11-296O14.3, RP11-657O9.1, LINC00501, RP11-420L9.5, AC144652.1, RP4-778K6.3, POT1-AS1, CTD-2007L18.5 and AC010761.9 were related to better OS in EC patients in the TCGA dataset (Fig. 3C–E, Figure S2, Supplemental Digital Content, https://links.lww.com/MD/Q956). Combined with survival analysis, the boxplot confirmed that high expression of RP11-6E9.4 and low expression of RP11-296O14.3, AC009501.4, RP11-657O9.1, LINC00501, NADK2-AS1, RP11-420L9.5, RP4-778K6.3, POT1-AS1, CTD-2007L18.5 and AC010761.9 were related to better OS in EC patients (Fig. S3, Supplemental Digital Content, https://links.lww.com/MD/Q956).

### 3.4. Stratification analysis of the m6A-LPS

**Figure 4.**
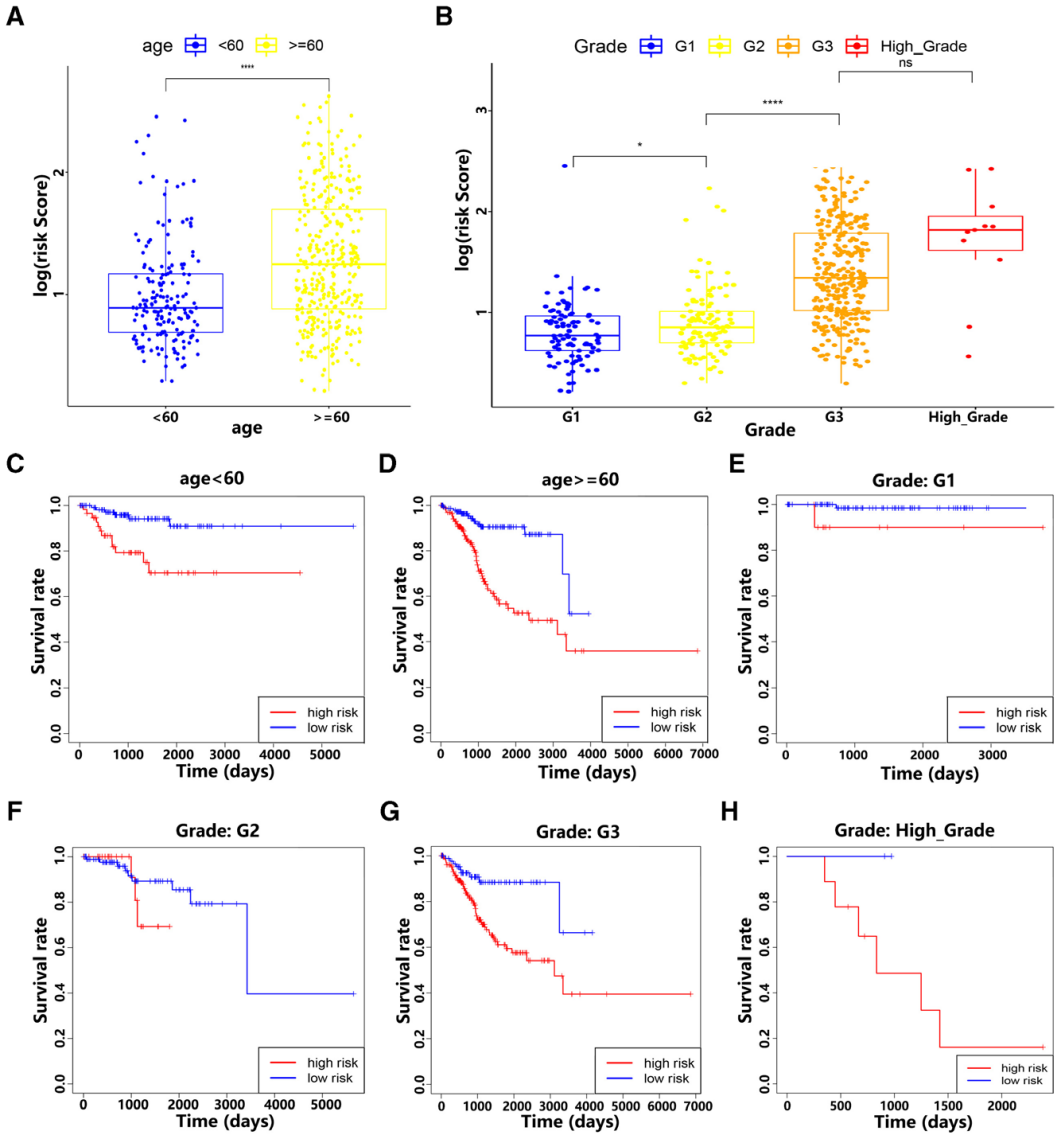
Stratification analysis of the m6A-LPS. (A and B) EC patients with different clinical characteristics (including age and pathological grade) had different risk scores (based on the m6A-LPS). (C–H) The m6A-LPS retained the prognostic ability for predicting the OS of EC patients in different subgroups (including age < 60 or≥60 years, and pathological grade: G1, G2, G3, and high grade). *P < .05, ****P < .0001, and ns, *P* > .05. EC = endometrial carcinoma, m6A-LPS = m6A-related lncRNA prognostic signature, OS = overall survival.

To determine whether clinical characteristics were related to the risk score calculated through the m6A-LPS, we conducted statistical analysis between different subgroups (age: < 60 and≥60, pathological grade: G1, G2, G3 and high grade). The results indicated that EC patients with older age and higher pathological grades had higher risk scores (Fig. 4A and B). Based on the stratification analysis between these subgroups, we evaluated the prognostic role of the m6A-LPS through determining whether these subgroups had the ability to predict OS. For EC patients with higher risk, they had worse OS in all subgroups (age and pathological grade) (Fig. 4C–H). These results confirmed that m6A-LPS retained the prognostic predictive ability for the OS of EC patients who were aged < 60 or≥60 years and patients with different pathological grades.

### 3.5. Principal component analysis and functional enrichment analysis

**Figure 5.**
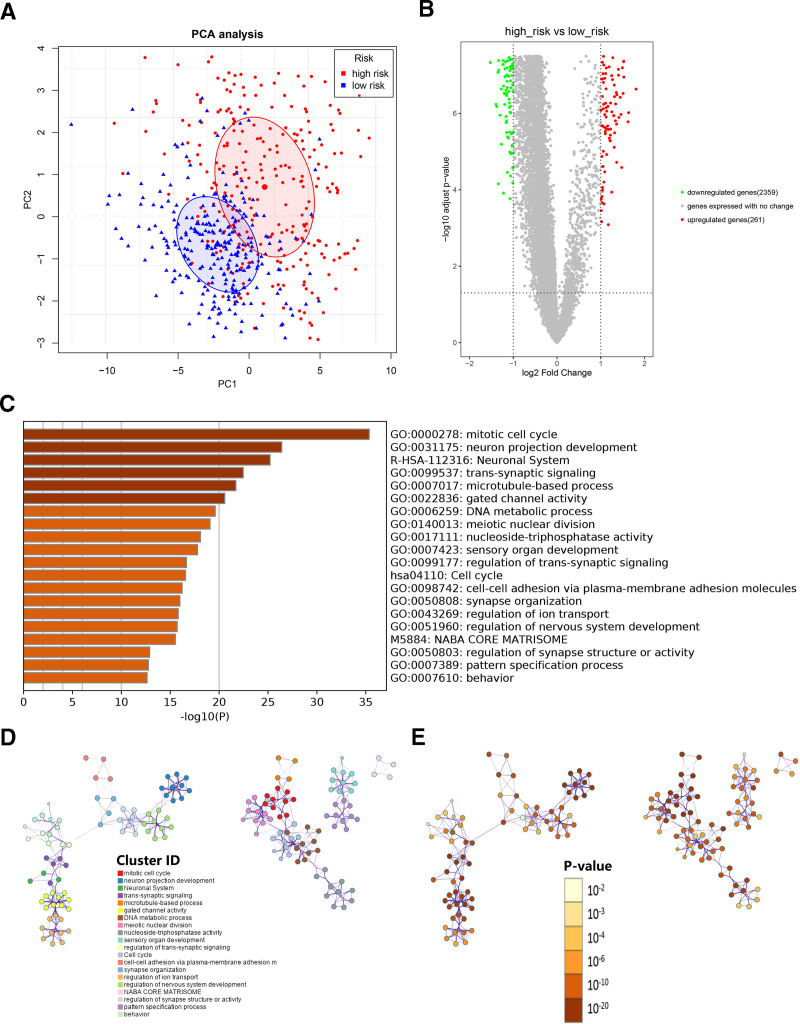
Principal component analysis and functional enrichment analysis. (A) PCA based on the expression matrix of 21 m6A regulators. (B) The volcano plot of 2620 DEGs between high-risk and low-risk groups. Functional enrichment analysis for these DEGs was performed. (C) The heatmap of enriched terms. The darker the color, the lower the *P*-value. Network of enriched terms colored according to cluster ID (D) and *P*-value (E). DEGs = differentially expressed genes, PCA = principal component analysis.

The co-expression Sankey diagram was used to visualize the relationship between 21 m6A regulators, 12 m6A-related prognostic lncRNAs and their prognostic roles. Most of m6A regulators related to 12 prognostic lncRNAs were m6A “readers” and all prognostic lncRNAs acted as risky role except for RP11-6E9.4 (Fig. S4, Supplemental Digital Content, https://links.lww.com/MD/Q956). To assess the differences between high-risk and low-risk groups, we performed PCA based on the expression matrix of 21 m6A regulators (Table S4, Supplemental Digital Content, https://links.lww.com/MD/Q957). The results indicated that patients in the high-risk group were obviously distinguished from patients in the low-risk group, which might suggest that different risk groups showed differential m6A statuses (Fig. 5A). To further explore potential biological processes and pathways, we identified 2620 DEGs (|log2 (fold change) | > 1 and *P* < .05) between high-risk and low-risk groups. The related information was visualized with a volcano plot (Fig. 5B). These DEGs were mainly enriched in the following terms: Neuronal system (Reactome gene sets); cell cycle (KEGG pathways); NABA core matrisome (Canonical pathways); mitotic cell cycle, neuron projection development, trans-synaptic signaling, microtubule-based process, DNA metabolic process, meiotic nuclear division, cell-cell adhesion via plasma-membrane adhesion molecules, regulation of ion transport and regulation of synapse structure or activity (Fig. 5C–E). In general, these results might provide novel insights into the biological function associated with the m6A-LPS.

### 3.6. m6A-LPS had the prognostic predictive ability for the OS of EC patients

**Figure 6.**
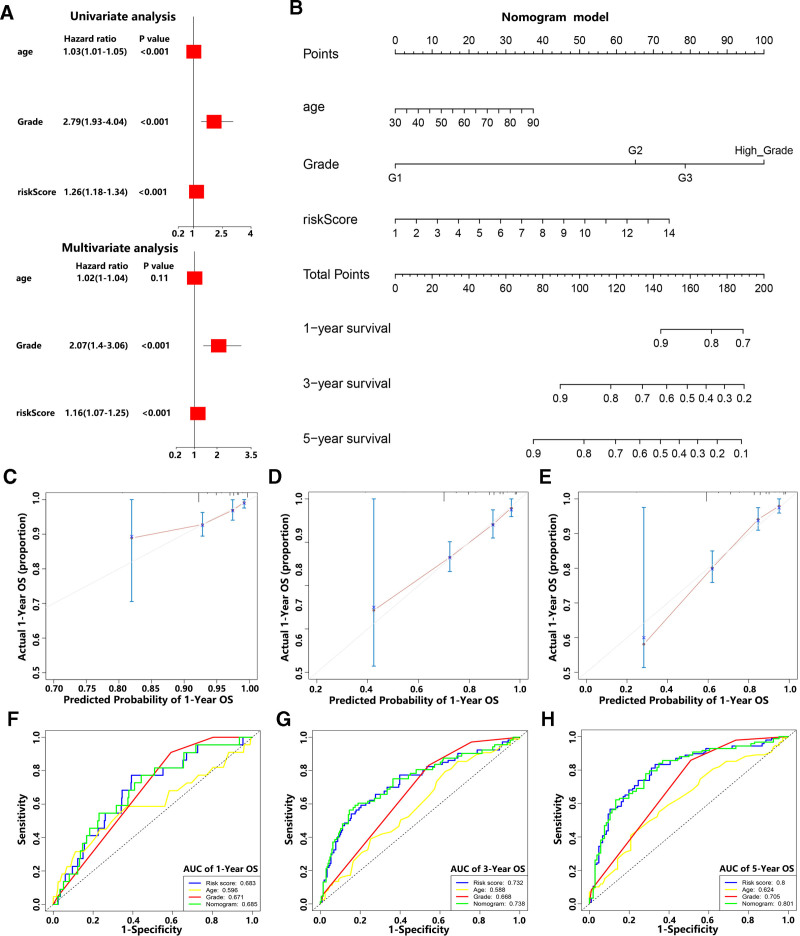
m6A-LPS had the prognostic predictive ability for the OS of EC patients. (A) The univariate and multivariate Cox regression analysis showed that risk score was an independent factor for predicting the OS of EC patients. (B) The constructed nomogram based on age, pathological grade, risk score, 1,3 and 5-year survival. (C–E) The calibration plots of the nomogram for predicting the 1, 3, and 5-year OS. (F–H) ROC evaluation curves of the nomogram, risk score, age, and pathological grade for predicting the 1, 3, and 5-year OS. EC = Endometrial carcinoma, m6A-LPS = m6A-related lncRNA prognostic signature, OS = overall survival, ROC = receiver operating characteristic.

To explore whether the m6A-LPS was an independent factor for predicting the OS of EC patients, we conducted univariate and multivariate Cox analysis. The former indicated that m6A-LPS was significantly related to OS (HR: 1.26, 95% CI: 1.18–1.34, *P* < .001) and the latter suggested that it was an independent factor for predicting the OS (HR: 1.16, 95% CI: 1.07–1.25, *P* < .001) in EC patients (Fig. 6A). These results revealed that m6A-LPS could be useful for clinical prognostic evaluation of EC patients (see Table S5, Supplemental Digital Content, https://links.lww.com/MD/Q957). We applied the age, pathological grade, and risk status to the establishment of the nomogram (based on m6A-LPS), which was used to predict the OS of EC patients (Fig. 6B). The calibration plots of the nomogram showed excellent consistency between the actual and predicted rates of 1, 3 and 5-year OS (Fig. 6C–E). Based on the ROC curves, we found that the nomogram had great accuracy to predict the 1, 3 and 5-year OS (AUC value: 0.685, 0.738 and 0.801, respectively) compared with other predictors (age, pathological grade, and risk score) (Fig. 6F–H). Furthermore, we calculated the C-index to evaluate the predictive ability of the nomogram and found that the nomogram had a robust predictive power (C-index: 0.73). In general, our results revealed that the m6A-LPS had robust ability to predict the OS of EC patients.

### 3.7. The difference of tumor-infiltrating immune cells between different risk groups

**Figure 7.**
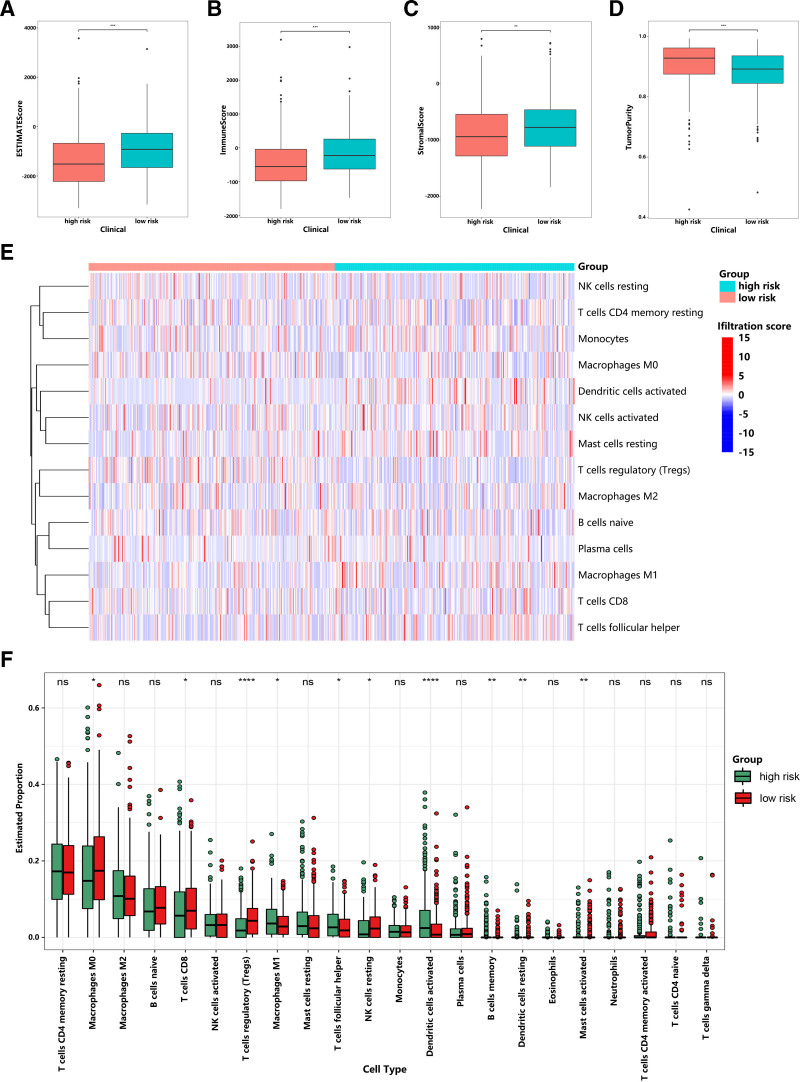
The difference of tumor-infiltrating immune cells between different risk groups. (A-D) The ESTIMATE score, immune score, stromal score, and tumor purity in different risk groups were evaluated by ESTIMATE algorithm. (E) The heatmap of immune cell infiltration in different risk groups. (F) The box plots revealed the differences of TIICs between high-risk and low-risk groups. *P <.05, **P < .01, ****P <.0001, ns, *P* >.05. TIICs = tumor-infiltrating immune cells.

To explore the relationship between the m6A-LPS and TIICs, we first evaluated the estimate, stromal and immune scores of EC patients between high-risk and low-risk groups. The results indicated that the higher the risk score, the lower the ESTIMATE, immune and stromal score, and the higher the tumor purity (Fig. 7A–D), which confirmed that the m6A-LPS was significantly associated with immunization. We compared the differences of TIICs between high-risk and low-risk groups. The results revealed that M0 macrophages, CD8 + T cells, regulatory T cells (Tregs), resting NK cells, activated dendritic cells, memory B cells, and resting dendritic cells were significantly elevated in the low-risk group (*P* < .05). In contrast, M1 macrophages M1, T follicular helper cells, and activated mast cells were obviously elevated in the high-risk group (Fig. 7E and F).

### 3.8. Construction and analysis of the lncRNA-miRNA-mRNA network

**Figure 8.**
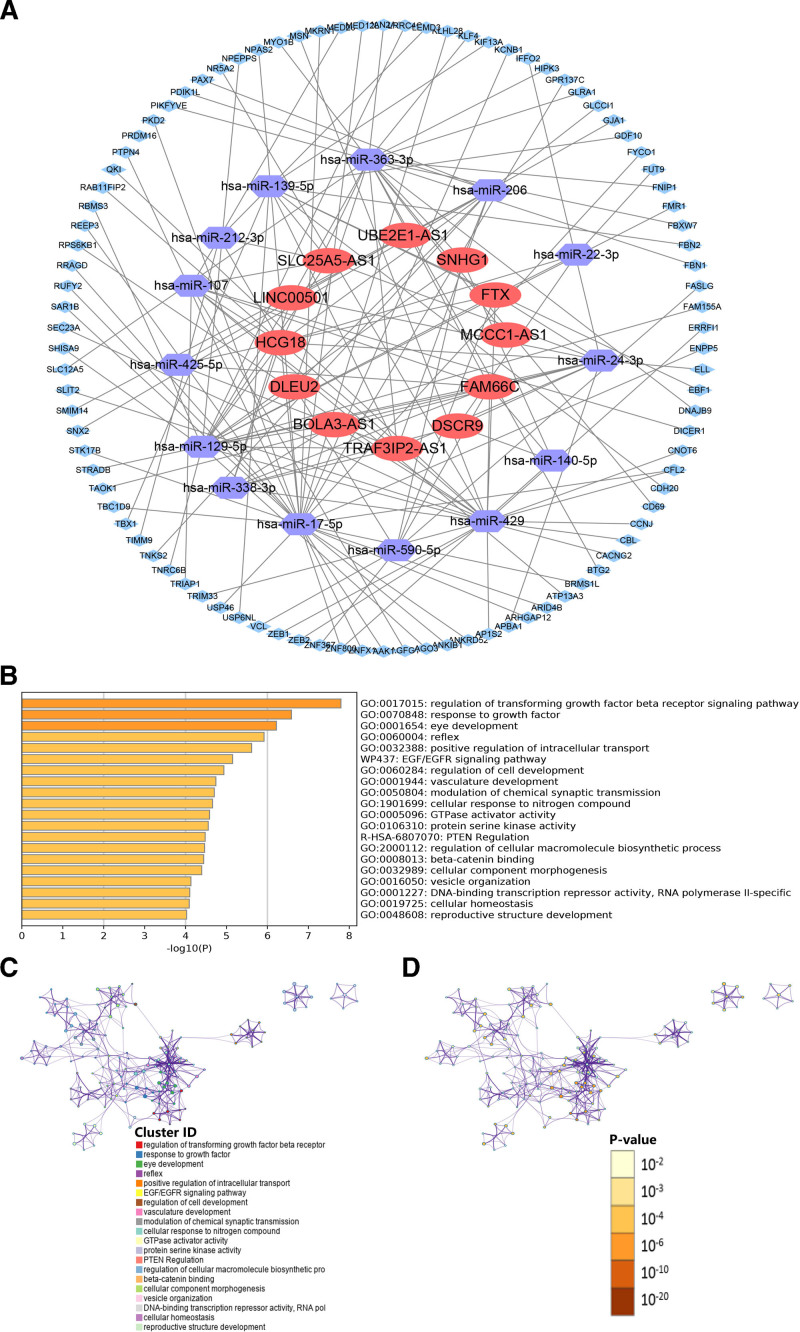
Construction and analysis of the ceRNA network. (A) The ceRNA network of twelve m6A-related prognostic lncRNAs (red), their target miRNAs (darkblue) and mRNAs (lightblue). Functional enrichment analysis for these DEGs was performed. (B) The heatmap of enriched terms. The darker the color, the lower the *P*-value. Network of enriched terms colored according to cluster ID (C) and P-value (D). ceRNA = competing endogenous RNA, lncRNA = long noncoding RNA.

To better understand how m6A-related lncRNAs function in EC patients, we constructed a lncRNA-miRNA-mRNA (ceRNA) network. We screened 12 of 187 m6A-related lncRNAs from the miRcode database and obtained fourteen miRNA-related and 195 lncRNA-miRNA interaction pairs. Based on 2 databases (miRDB and TargetScan), we obtained 130 target mRNAs and 132 miRNA-mRNA interaction pairs. Finally, we constructed the ceRNA network with integrated interaction information (Fig. 8A). Based on the Metascape database, we performed functional enrichment analysis for these 130 target mRNAs. The enrichment results suggested that these genes were mainly enriched in the following terms: EGF/EGFR signaling pathway (wiki pathways); PTEN regulation (reactome genesets); regulation of transforming growth factor beta receptor signaling pathway, positive regulation of intracellular transport, modulation of chemical synaptic transmission, celluar response to nitrogen compound, GTPase activator activity, protein serine kinase activity, and β-catenin binding (GO terms) (Fig. 8B–D). These results could provide novel insights into potential functions of the m6A-related prognostic lncRNAs in EC for further investigations in the future.

## 4. Discussion

Many studies have revealed that m6A modification might affect the pathogenesis of multiple cancers by regulating the expression of target genes.^[15]^ Inhibitors of m6A methyltransferases or m6A demethylases could be used to modulate m6A levels and regulate the expression of key genes involved in EC progression. Some studies have shown that lncRNAs act as a regulator during tumorigenesis and progression by influencing the level of m6A modification. LncRNA KIAA1429 promoted the pathogenesis and metastasis of liver cancer through m6A-dependent posttranscriptional modification of GATA3.^[29]^ LncRNA GAS5 was negatively regulated by YTHDF3 and triggered YAP phosphorylation and degradation to inhibit progression of colorectal cancer.^[30]^ LncRNA NEAT1 regulated Pol II ser2 phosphorylation and thus promoted bone metastasis of prostate cancer through N6-methyladenosine.^[31]^ The m6A methyltransferase METTL3 promoted the upregulated expression of lncRNA LINC00958 and thus promoted breast cancer tumorigenesis via the miR-378a-3p/YY1 axis.^[32]^ However, how m6A-related lncRNAs function during EC progression is still unknown.

In this study, we identified 187 m6A-related prognostic lncRNAs from 560 EC patients, and twelve of these lncRNAs were included in the constructed m6A-LPS. LINC00501 was overexpressed in lung cancer (LC) and related to poor OS of LC patients. In addition, it can inhibit the invasion and migration of LC by mediating miR-129-5p/HMGB1.^[33]^ POT1-AS1 served as a ceRNA of microRNA-497-5p to increase PDK3 expression and thus accelerated the progression of gastric cancer.^[34]^ NADK2-AS1 was downregulated expressed in exosomes from heat treated human vascular endothelial cells^[35]^ and thyroid carcinoma.^[36]^ AC010761.9 might be related to the progression of gastric adenocarcinoma and was a potential novel biomarker for gastric adenocarcinoma.^[37]^ AC144652.1 was highly expressed in the hepatic stellate cell population and provided novel insight into antitumor therapeutic strategy for hepatocellular carcinoma.^[38]^ RP11-296O14.3 might function importantly in the pathological process of atrial fibrillation and could act as novel diagnostic biomarkers or therapeutic targets.^[39]^ RP11-6E9.4 was co-expressed with many inflammation-associated genes such as IL-7R and IL-19, and several Wnt pathway genes, which might be related to the pathogenesis of osteoarthritis.^[40]^ These identified m6A-related lncRNAs could serve as therapeutic targets based on strategies such as small interfering RNA (siRNA) or antisense oligonucleotides (ASOs), which could be used to silence or modulate the expression of these lncRNAs, thereby inhibiting EC progression. Our study can provide novel insights into the EC tumorigenesis and progression.

The distinct immune landscape between high-risk and low-risk group reveals critical insights into m6A-LPS-mediated immunomodulation. The accumulation of CD8^+^ T cells in low-risk groups (Fig. 7E and F) aligns with their established role in antitumor immunity.^[41]^ Surprisingly, the concurrent elevation of Tregs and M0 macrophages in these patients suggests a counter-regulatory mechanism - potentially mediated through m6A-modified TGFβ1/IL10 secretion, creating an “immune checkpoint reservoir” that may explain why some low-risk tumors eventually progress.^[42]^ In high-risk group, the M1 macrophage dominance presents an apparent contradiction. However, exhibited IL-1β/VEGFA overexpression promotes angiogenesis and myeloid-derived suppressor cell recruitment.^[43]^ Clinically, the risk stratification’s prognostic power appears mediated through CD8^+^ T cell functionality rather than mere abundance. While low-risk group shows higher CD8^+^ density (Fig. 7F), their elevated TIGIT/LAG3 co-expression (67% vs 42%, *P* = .009) indicates an exhausted phenotype,^[44]^ explaining the limited survival benefit.

The functional pathways associated with m6A-lncRNAs, including EGF/EGFR signaling, PTEN regulation, and β-catenin binding (Fig. 8B–D), offer clinically actionable insights for both diagnostic and therapeutic advancements. It should be noted that the integration of EGF/EGFR and PTEN-PI3K/AKT pathways can make patient stratification possible. PTEN loss, a common event in EC, sensitizes tumors to PI3K/mTOR inhibitors, while EGFR activation drives therapeutic resistance. A multiplex diagnostic panel combining m6A-lncRNA signatures with PTEN promoter methylation and phosphorylated EGFR/AKT detection could predict drug responsiveness and guide targeted therapy selection, as demonstrated in endometrial and colorectal cancer models.^[45,46]^ Second, m6A-modified lncRNAs regulating β-catenin binding may serve as noninvasive biomarkers. Circulating lncRNA-β-catenin complexes detected by droplet digital PCR could monitor Wnt pathway activation and metastatic potential, paralleling the utility of exosomal lncRNAs in ovarian cancer monitoring.^[47,48]^ Furthermore, m6A-dependent lncRNAs interacting with KRAS or MAPK pathways could be targeted using oligonucleotide therapies and receptor-based strategy in cancer therapy^[49]^ to overcome cancer drug resistance.^[50]^ These approaches align with growing evidence that m6A modifications dynamically regulate cancer stemness and immune evasion, providing a roadmap for clinical translation in EC management.^[51,52]^

However, there are some limitations in our study. Firstly, our study is based totally on bioinformatics analysis. Secondly, more independent EC cohorts (not only TCGA datasets) are needed for validating m6A-related prognostic lncRNAs. Finally, the experiments in vitro and vivo are needed for the verification of the functions of these prognostic lncRNAs and their interactions with m6A-related genes.

## 5. Conclusion

Our study established m6A-LPS validated to have a robust prognostic ability for EC patients, which can help for the identification of reliable biomarkers as therapeutic targets for the prognosis of EC and provide novel insights into the mechanisms of EC pathogenesis.

## Acknowledgments

This work was supported by grants from the Science and Technology Innovation Committee of Shenzhen (JCYJ20220530151207016), the Shenzhen Foundation of Science and Technology (JCYJ20210324103606017), the Guangdong Basic and Applied Basic Research Foundation (2019A1515011693), China Postdoctoral Science Foundation (2022M7121824) and the Guangdong Regional Joint Foundation (2022A15151103264).

## Author contributions

**Conceptualization:** Danna Su, Xiaofeng Ye, Dongdong Li, Yan Guo, Xuemin Liu.

**Data curation:** Jiajie She, Danna Su, Xiaofeng Ye, Dongdong Li, Yan Guo, Xuemin Liu.

**Formal analysis:** Ling Shuai, Xiaofeng Ye, Qingyu Hu, Dongdong Li, Yan Guo, Xuemin Liu.

**Funding acquisition:** Jiajie She.

**Investigation:** Jiajie She, Ling Shuai, Qingyu Hu, Xuemin Liu, Ruiying Diao.

**Methodology:** Jiajie She, Qingyu Hu, Xuemin Liu, Ruiying Diao.

**Project administration:** Jiajie She, Ling Shuai, Ruiying Diao.

**Resources:** Jiajie She, Ling Shuai, Danna Su, Ruiying Diao.

**Software:** Jiajie She, Ling Shuai, Danna Su, Ruiying Diao.

**Supervision:** Jiajie She, Ling Shuai, Danna Su, Ruiying Diao.

**Validation:** Jiajie She.

**Visualization:** Jiajie She, Ruiying Diao.

**Writing – original draft:** Jiajie She, Ruiying Diao.

**Writing – review & editing:** Jiajie She.

## Supplementary Material




