# Supplementary material for: N6-methylandenosine-related lncRNAs have the prognostic predictive ability for patients with endometrial cancer
Source: Medicine (Baltimore). 2025 Dec 19;104(51):e46657. doi: 10.1097/MD.0000000000046657 (PMC12727374; doi:10.1097/MD.0000000000046657)

**Supplementary Figure 1. The heatmap of 187 m6A-related prognostic lncRNAs.**

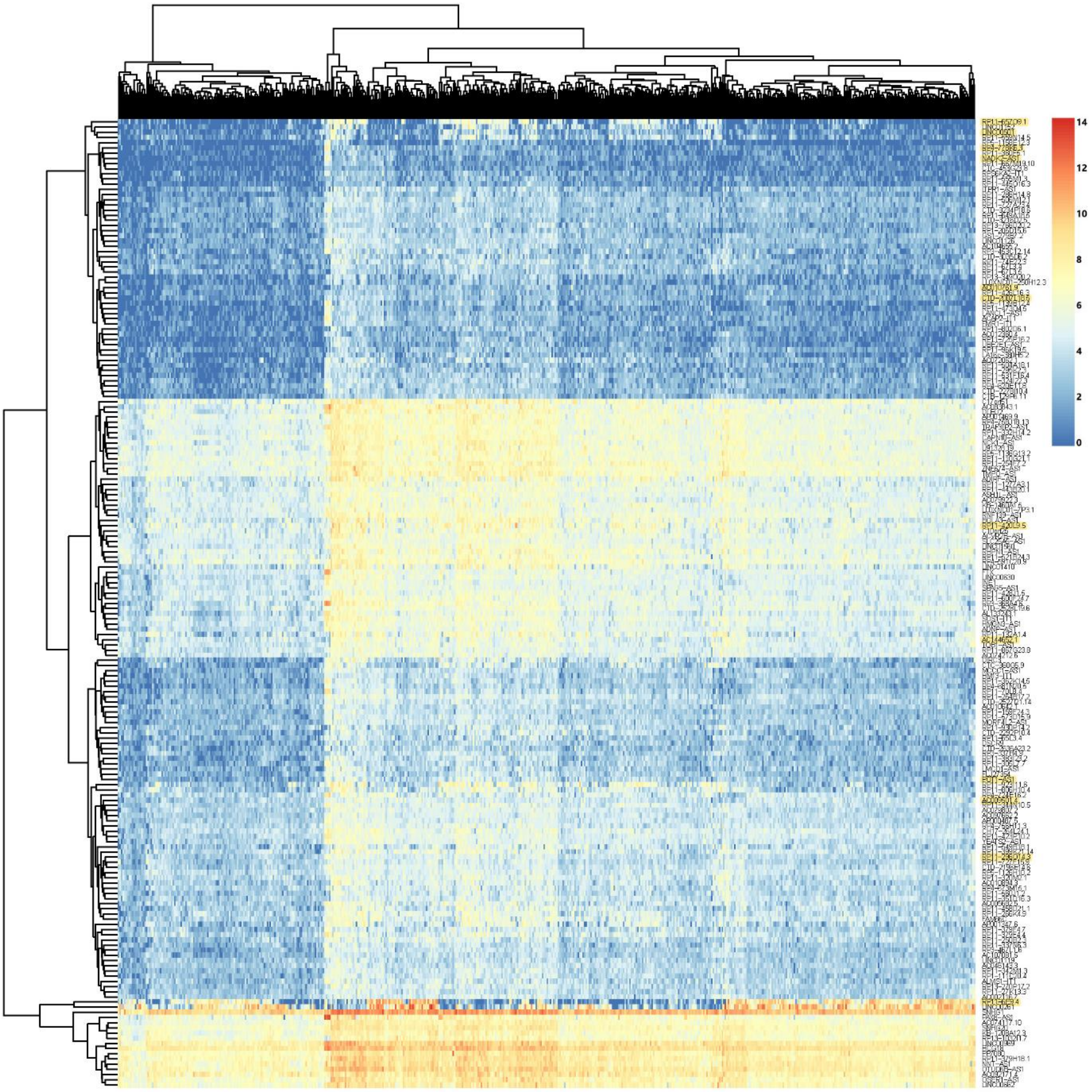

**Supplementary Figure 2. Kaplan–Meier curves.**

(**A-I**) Kaplan–Meier curves revealed the relationship between the expression levels of six m6A-related prognostic lncRNAs and OS.

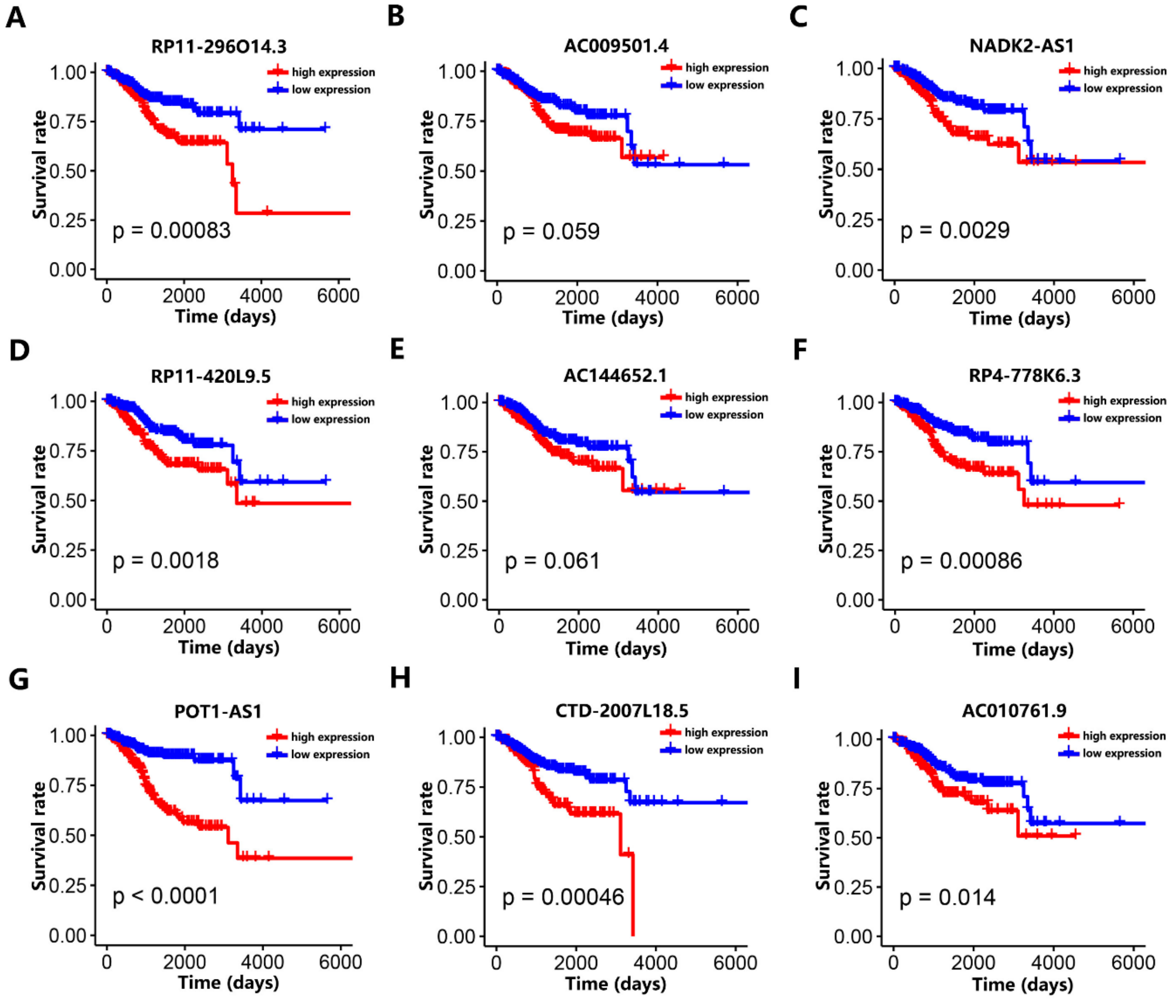

**Supplementary Figure 3. The box plots.**

(A-L) The box plot showed the relationship between the expression levels of twelve m6A-related prognostic lncRNAs and survival status. *p < 0.05, **p < 0.01, ***p < 0.001, ****p < 0.0001 and ns, p > 0.05.

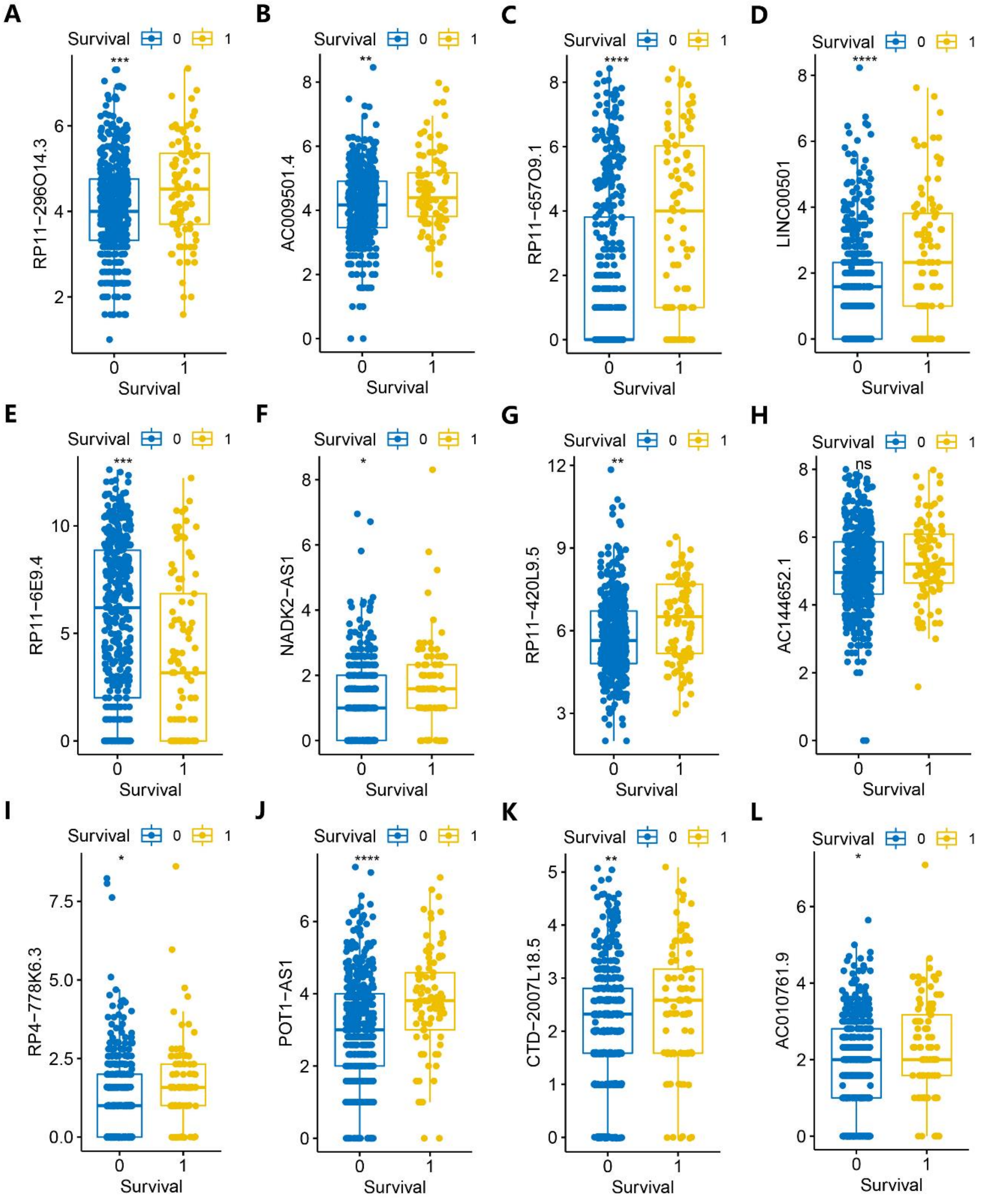

**Supplementary Figure 4. Co-expression Sankey diagram.**

The co-expression Sankey diagram revealed the relationship between m6A regulators, m6A-related prognostic lncRNAs and risk types.

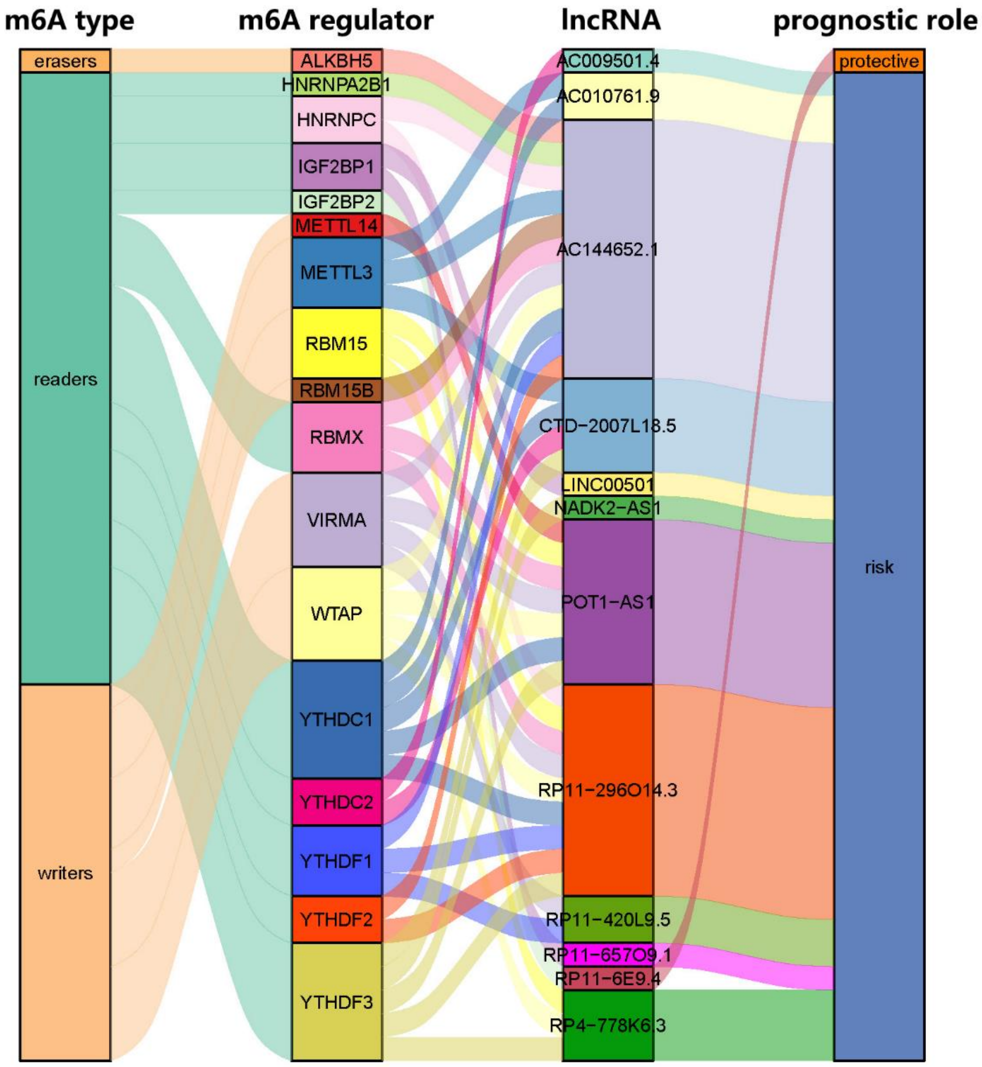

Supplement: Supplementary file 2 [file medi-104-e46657-s002.pdf]
